# 23.3 DEVELOPMENTAL TRAJECTORIES OF SCHIZOPHRENIA-RELEVANT ABNORMALITIES IN A MOUSE MODEL OF 22Q11.2 DELETION SYNDROME

**DOI:** 10.1093/schbul/sby014.093

**Published:** 2018-04-01

**Authors:** Mariasole Ciampoli, Marco Armando, Francesco Papaleo

**Affiliations:** 1 Istituto Italiano di Tecnologia; 2 Hospital Bambino Gesu

## Abstract

**Background:**

The hemizygous genetic deletion in the 22q11.2 locus causes a syndrome (22q11DS) characterized by developmental social and intellectual disabilities, high prevalence of attention deficit hyperactivity disorder (ADHD; ≈37%) during childhood and schizophrenia (≈41%) in adulthood. Although this peculiar behavioral alterations, the specific brain and molecular factors influencing these developmental trajectories are still unknown. Preclinical animal studies could help to disentangle these mechanisms. However, no studies in animal models had so far checked the impact of the 22q11.2 microdeletion in behavioral phenotypes from birth to adolescence, to adulthood.

**Methods:**

We used LgDel mutant mice that carry the same 1.5 Mb deletion of the human 22q11.2DS. In parallel, we also used patients with 22q11.2DS.

**Results:**

We first unraveled in mice altered startle responses at pre-pubertal ages (postnatal day PND 14) that ameliorated in early development from PND 19. Moreover, sensorimotor gating deficits started to appear as early as PND 19 lasting throughout adulthood. Motor coordination assessed with the Rotarod Test, instead revealed in LgDel mice motor deficits in pre-pubertal period (PND 15–16), that disappeared from adolescence (PND 35). Next, in an implemented 5-Choice Serial Reaction Time Task, we found that LgDel adolescent mice showed selective higher distractibility than controls as it has been shown in patients with schizophrenia. All these developmental behavioral alterations were accompanied by selective altered maturation of the prefrontal cortex, as demonstrated by parallel studies in mice and humans.

**Discussion:**

Overall, our experiments are starting to elucidate how clinically-relevant genetic alterations can influence the developmental trajectories of behavioral phenotypes through an altered maturation of the prefrontal cortex. This will be important in the context of the development of early diagnosis and preventive intervention.

